# High-Sensitivity Terahertz Biosensor Based on a Multi-Layer Hybrid Structure Consisting of a Defect Mode and Graphene

**DOI:** 10.3390/bios15100702

**Published:** 2025-10-17

**Authors:** Hai Hu, Shiying Mo, Yangbao Deng, Zhengchun Zhao

**Affiliations:** All-solid-state Energy Storage Materials and Devices Key Laboratory of Hunan Province, School of Information and Electronic Engineering, Hunan City University, Yiyang 413000, China; huhai@hncu.edu.cn (H.H.); moshiying@hncu.edu.cn (S.M.); zhaozhengchun@hncu.edu.cn (Z.Z.)

**Keywords:** terahertz biosensor, graphene, defect mode

## Abstract

A high-sensitivity terahertz (THz) biosensor is proposed in this paper based on a multi-layer hybrid structure consisting of a defect mode and graphene with a truncation layer. This biosensor is based on symmetrical Bragg reflectors with a defect layer and graphene with a truncation layer, which effectively comprise a multi-layer hybrid resonance excitation structure. The high sensitivity of this biosensor is developed through defect mode resonance, and the resonance reflection peak is made sharper and more sensitive by using graphene with a truncation layer. After testing and analysis, the sensitivity of this biosensor structure is greatly affected by the refractive index and thickness of the sensing medium. By setting parameters appropriately, the composite structure can be used as both a liquid biosensor and a gas biosensor, the maximum sensitivity of which can surpass 2000°/RIU, while an FOM value of 22,500 RIU^−1^ can be achieved. At the same time, when the refractive index of the liquid sensing medium changes to 0.01 relative to water (the same applies to changes in the gas sensing medium), the sensitivity of this structure still exceeds 600°/RIU, demonstrating that this biosensor has advantages including high sensitivity, a high FOM, wide applicability, and slow sensitivity attenuation. Therefore, the sensing scheme proposed in this paper has potential application prospects in the field of biosensing based on micro/nanostructures due to its simple structure, low requirements for processing conditions, and high sensitivity.

## 1. Introduction

Optical biosensors carry out the conversion of biological information into optical signals, transforming invisible biological phenomena into visible or measurable optical phenomena and converting biological signals into optical signals that can conveniently be used for measurement and quantification [1]. They provide non-contact and non-destructive measurements, display strong anti-interference ability, and have high sensitivity [2], and they have been widely used in various biological detection fields, including environmental monitoring [3], food safety [4], pathogen detection [5], biomedicine research [6], small molecule detection [7], etc. Micro/nanostructures, due to their easy integration and small size, have gradually become popular among researchers working with biosensors in recent years, and many related schemes have been proposed, including photonic crystals [8], microring cavity structures [9], carbon nanotubes [10], THz plasmas [11], toroidal dipole resonance [12], etc. Based on the sensitivity of surface plasmon resonance (SPR) to subtle changes in the external environment, SPR has been widely studied and applied in the field of micro/nanostructure optical sensing [13,14]. Through research and analysis of new materials and mechanisms, various schemes of optical biosensors incorporating SPR have been proposed by researchers with the aim of achieving the research goals of high sensitivity, dynamic controllability, and simple structure [15,16,17].

The defect mode of one-dimensional photonic crystals is an intriguing physical phenomenon that has attracted widespread attention from researchers and that plays an important role in fields such as optical communication, sensors, and optical devices. When a defect is introduced into a one-dimensional photonic crystal structure by altering the refractive index or thickness of a specific layer, it disrupts its periodicity, potentially creating a defect mode. The energy of the defect mode field is typically localized at the defect site, characterized by a narrow frequency range and high light intensity. This property facilitates the construction of high-quality-factor photonic crystal microcavities and enables high-sensitivity, high-resolution biological sample sensing. For example, Wang et al. designed a high-quality-factor slot photonic crystal nanobeam cavity structure based on SOI material [18]; Yang et al. proposed a silicon-based highly integrated one-dimensional photonic crystal nanobeam cavity sensor array structure [19]; Stanley proposed a photonic crystal microring resonator biosensor through experiments [9]; Jena et al. proposed a new one-dimensional photonic crystal (1D PC) containing moderately doped silicon semiconductor for THz light-based thermal sensors with a defect mode [20]; and Ye et al. used a composite structure of graphene and a one-dimensional photonic crystal to create highly sensitive and tunable biosensors in the terahertz band [21]. Tang et al. studied a multi-layer structure biosensor in the terahertz band, placing the sensing medium between 1D PC and graphene to enhance optical high sensitivity through optical resonance excitation of OTS [22]. Liu et al. designed a hybrid structure with a built-in defect layer based on the OTS structure, which coupled the Tamm surface plasmon mode and the defect mode to obtain high-sensitivity terahertz biosensors [23]; Bao et al. used the same hybrid structure but replaced graphene with Dirac material, and also obtained high-sensitivity terahertz biosensors based on mode coupling between the Tamm surface plasmon mode and the defect mode [24].

As a typical two-dimensional material, graphene has attracted significant attention from researchers in the biosensor field in recent years due to its metallic properties, ultra-thin structure, and excellent photoelectric characteristics [25,26]. Additionally, surface-localized modes, such as Bloch surface waves (BSWs), propagating at the interface between a continuous medium and a truncated periodic full dielectric structure represent a case of local field enhancement [27]. The truncation of one-dimensional photonic crystals at the surface disrupts their lattice’s periodicity, leading to the formation of a localized electromagnetic field on the surface of the truncation layer. For example, Zheng et al. investigated near-infrared biosensors using a BSW structure based on Dirac semimetals to meet high sensitivity requirements [28]. On this basis, we theoretically propose a high-sensitivity optical biosensor based on a multi-layer hybrid structure combining a defect layer and graphene with a truncation layer. This biosensor utilizes a symmetrical Bragg reflector composed of one-dimensional photonic crystals with an embedded defect layer, which effectively forms a multi-layer hybrid resonant excitation structure. This structure retains the characteristics of the defect mode [23] while incorporating the local field enhancement properties of the truncation layer [28], which generates a resonance reflection peak in the terahertz frequency band. By utilizing the characteristics of graphene and the local field enhancement of the truncation layer, the resonance reflection peak becomes sharper and more sensitive, thereby achieving high biosensor sensitivity. Testing and analysis reveal that factors significantly affecting the sensitivity of this structure include the refractive index and the thickness of the sensing medium. In order to expand the application potential of this work, we simultaneously tested the biosensor under gas and liquid conditions through comparative analysis. After structural optimization and parameter adjustment, the biosensor maintains sensing performance comparable to liquid scenarios under gas conditions, and the refractive index sensitivity can reach over 2000°/RIU, while an FOM value of 22,500 RIU^−1^ can be achieved. This biosensor, with a micro/nanostructure, offers advantages such as a simple structure, strong adaptability to different scenarios, high sensitivity, a high FOM, and slow sensitivity attenuation. We believe it holds great potential for application in this field.

## 2. Theoretical Models and Methods

**Figure 1:**
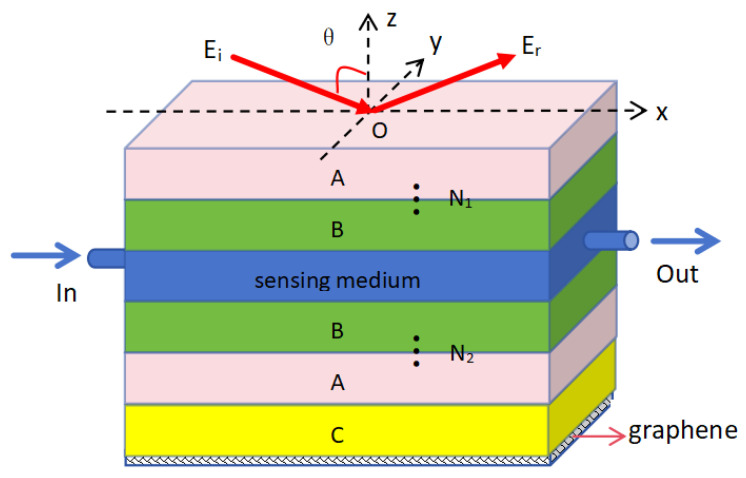
Schematic diagram of a terahertz biosensor based on a multi-layer hybrid structure with a defect mode incorporating graphene. From top to bottom, the structure consists of Photonic Crystal 1, a sensing medium layer, Photonic Crystal 2, a truncation layer, and graphene.

On the basis of a typical 1D PC multi-layer structure, we consider a hybrid structure consisting of a built-in defect layer, symmetrical Bragg reflectors (DBRs), and graphene with a truncation layer. The structure is divided into five main parts from top to bottom, as shown in Figure 1. Photonic Crystal 1 and Photonic Crystal 2 have similar structures with symmetrical distributions; both are DBR structures composed of two alternating dielectrics, A and B, with periods of $N_{1}$ and $N_{2}$, respectively. Their refractive indexes and thicknesses are represented as $n_{a}$, $n_{b}$, $d_{a}$, and $d_{b}$, respectively. A sensing medium layer with refractive index $n_{s}$ and thickness $d_{s}$ is placed between the two photonic crystals. In order to meet the application requirements of biosensors in practical scenarios, input and output ports are integrated into the sensing medium layer, serving as a circulation pool for liquid/gas. This configuration enables the formation of a defect mode within the photonic crystals. Additionally, graphene is positioned at the bottom of the structure, separated from Photonic Crystal 2 by a truncation layer, creating a plasmonic-like mode and ultimately forming a multi-layer hybrid resonant excitation structure.

We assume that the incident light is incident from the air above the structure at an angle of *θ*, and the terahertz band is selected in the structure with a center wavelength of $\lambda_{c}$ = 300 μm. The refractive index and thickness of dielectric A are set as $n_{a}$ = 2.35 and $d_{a}$ = 31.12 μm, respectively, which can be achieved using glass in actual optical materials. In the same way, the refractive index and thickness of dielectric B are set as $n_{b}$ = 1.37 and $d_{b}$ = 44.9 μm, respectively, which can be achieved using MgF2 in actual optical materials. The refractive index and thickness of the truncation layer C are set to $n_{c}$=$n_{a}$=2.35 and $d_{c}$=0.5$d_{a}$, respectively. Currently, two photonic crystal periods are set to the same value: $N_{1}$ = $N_{2}$ = 12. Meanwhile, for the convenience of analysis, we do not take the impact of absorption by the biosensing layer into consideration. Firstly, analyze liquid sensing and set the refractive index and thickness of the sensing layer to $n_{s}$ = 1.33 and $d_{s}$ = 486.8 μm, respectively. Considering that the thickness of single-layer graphene is only 0.34 nm, we use conductivity to represent its photoelectric properties. However, during calculation, graphene can be divided into single-layer and multi-layer structural forms. After calculation, it was found that single-layer graphene meets the structural requirements in this paper. In addition, ignoring the external magnetic field and under the conditions of limited random phase approximation and a terahertz frequency range (where the inter-conductivity of graphene is much smaller than the intra-conductivity), considering only the single-layer embedding structure of graphene, its conductivity can be obtained as follows [29]:

(1) $$\sigma \approx \frac{ie^{2}E_{f}}{\pi \hbar^{2}(\omega +i/\tau )},$$

where e is the elementary charge constant, ℏ is the simplified Planck constant, ω is the angular frequency of the incident beam, *τ* is the relaxation time, and $E_{f}=\hbar v_{f}{\left(\pi n_{2D}\right)}^{1/2}$ is the Fermi energy. $v_{f}=10^{6}m/s$ is the Fermi velocity of the electron, and $n_{2D}$ is the carrier density. It can be inferred that the Fermi energy $E_{f}$ can be directly obtained through the carrier density $n_{2D}$, which can be controlled by the external voltage. Therefore, the surface conductivity of graphene can be flexibly controlled by the applied voltage, making its electrical conductivity characteristics tunable. In the following calculations, the Fermi energy of graphene is set to $E_{f}$ = 0.5 eV, and the relaxation time is set to *τ* = 1 ps.

In order to effectively measure the sensing performance in the next step, it is necessary to calculate the reflectivity of the entire structure using the mature and classic transmission matrix method [30]. As multi-layer hybrid resonant excitation structures can be excited in both TM and TE polarization modes, the transfer matrices between graphene and the dielectric (the truncation layer in this structure) can be expressed as follows [30]:

① TM polarization mode:

(2) $$D_{c->o}=\frac{1}{2}\left[\begin{array}{cc} 1+\eta_{Co}+\xi_{Co} & 1-\eta_{Co}-\xi_{Co}\\ 1-\eta_{Co}+\xi_{Co} & 1+\eta_{Co}-\xi_{Co} \end{array}\right],$$

where $\eta_{\mathrm{Co}}=\varepsilon_{C}k_{oz}/\varepsilon_{o}k_{Cz}$ and $\xi_{\mathrm{Co}}=\sigma k_{oz}/\varepsilon_{0}\varepsilon_{o}\omega$; here, $k_{oz}$ is the wave vector component of electromagnetic waves propagating in the lower air layer, and $k_{Cz}$ is the wave vector component of electromagnetic waves propagating in the dielectric C (the truncation layer).

② TE polarization mode:

(3) $$D_{c->o}=\frac{1}{2}\left[\begin{array}{cc} 1+\eta_{Co}+\xi_{Co} & 1-\eta_{Co}+\xi_{Co}\\ 1-\eta_{Co}-\xi_{Co} & 1+\eta_{Co}-\xi_{Co} \end{array}\right],$$

where $\eta_{\mathrm{Co}}=k_{oz}/k_{Cz}$ and $\xi_{\mathrm{Co}}=\sigma \mu_{0}\omega /k_{Cz}$.

Finally, we calculate the transmission matrix of the entire structure [30]. As mentioned earlier, the thickness of graphene is only 0.34 nm. Based on Formulas (2) and (3) and its conductivity characteristics, the graphene in this structure is considered as a boundary condition for calculation, rather than as a mono-layer. By substituting the above conditions into the propagation matrix formula, we can obtain

(4) $$\begin{array}{l} M={D_{i->A}(P_{A}D_{A->B}P_{B}D_{B->A})}^{N_{1}-1}P_{A}D_{A->B}P_{B}D_{B->S}P_{S}D_{S->B}\\ •{\left(P_{B}D_{B->A}P_{A}D_{A->B}\right)}^{N_{2}-1}P_{B}D_{B->A}P_{A}D_{A->C}P_{C}D_{C->o}, \end{array}$$

and then the reflection coefficient of the entire structure can be expressed as $r=M_{21}/M_{11}$ and the reflectance is $R={\left|r\right|}^{2}$.

In order to measure the performance indicators of biosensors, we regard the sensitivity as the core indicator. The sensitivity of this structure based on angle modulation is defined as [31]

(5) $$S=\frac{\Delta \theta }{\Delta n_{s}},$$

where Δθ represents the angle change of the reflection resonance peak and $\Delta n_{s}$ represents the change in the refractive index in the sensing medium layer.

Additionally, the quality factor (DA) is defined as DA = 1/FWHM (full width at half maxima), and the figure of merit (FOM) can be calculated using FOM = S·DA [22].

## 3. Results and Discussion

Compared to theoretical modeling, experimental validation involves more practical considerations, such as micro/nano-scale manufacturing, and requires relatively complex requirements. Therefore, the current study focuses on the theoretical aspect. It is important to note that there are mature experimental methods for sensing based on DBR-related structures. The implementation of such experimental work will be the main focus of our subsequent research stages.

**Figure 2:**
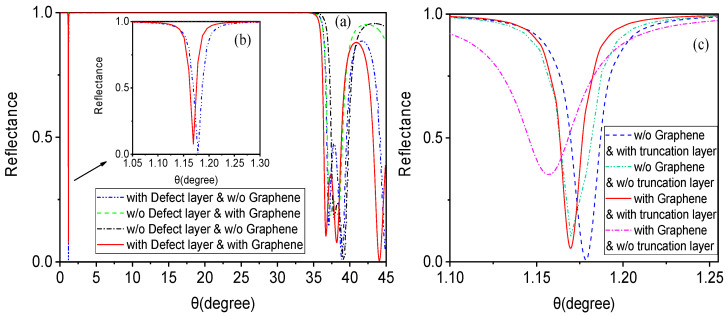
Influence of the defect layer and graphene on resonance excitation in the application case of the structure as a liquid biosensor. (**a**) Comparison chart of reflectance curves with incident angle for four different states: ① loaded with a defect layer but without graphene (blue dashed line), ② loaded with graphene but without a defect layer (green dashed line), ③ loaded with neither graphene nor a defect layer (black dashed line), ④ loaded with both graphene and a defect layer (red solid line). (**b**) Enlarged partial view of reflectance curves. (**c**) The influence of the truncation layer with graphene on the curves.

When discussing the effects of the defect layer and graphene with a truncation layer, it is important to note that biosensors (including Tamm state surface plasmon with or without graphene, as well as the multi-layer hybrid resonant excitation structure described in this paper) require observing trends in certain factors (such as the reflection peak of the refractive index) to measure their sensitivity. Therefore, we first focus on the correlation between the reflectivity of this structure and the incident angle to determine its sensitivity characteristics. For convenience of comparison and optical mechanism analysis, using the parameters discussed in the previous section, we plot comparison graphs of four state reflection curves in Figure 2. Figure 2a separately shows the impact of the defect layer and graphene on the reflection curve of this structure (with the truncation layer unchanged). Figure 2b provides an enlarged partial view of the reflectance curves. The resonant excitation structure mentioned here is insensitive to the incident angle, so its excitation conditions differ from SPR (where the reflection peak occurs at a position greater than the total reflection angle), and it can even satisfy excitation conditions at small angles. From Figure 2a, it can be observed that if only a distributed Bragg symmetric photonic crystal structure is used, the reflectance between 0° and 35° is nearly 1, indicating a typical photonic band gap (shown by the black dashed line). In this case, the entire structure functions as a Bragg reflector, with electromagnetic waves being almost unable to pass through, thus failing to meet the sensing requirements. On this basis, a sensing layer is embedded between the symmetrical photonic crystals to form a defect layer (as shown in Figure 1). The blue dashed line in Figure 2b reveals a distinct sharp reflection peak near 1.175°, a typical feature of a defect mode. Furthermore, when graphene and a defect layer are loaded simultaneously, resonance excitation modes will occur under appropriate parameter control, forming sharp resonance reflection peaks (as shown by the red solid line).

In order to better analyze the influence of the truncation layer with graphene on the curves, as shown in Figure 2c, the green dash-dotted line (with only a defect mode, loaded with neither graphene nor a truncation layer) displays the shape of the reflection peak corresponding to the defect mode. This reflection peak exhibits some noticeable deformations (particularly evident in the subsequent gas sensor analysis). On the basis of the defect mode, loading with a truncation layer clearly improves this deformation of the reflection peak, as shown by the blue dashed line in Figure 2c. Similarly, the red dotted line (indicating that both the defect mode and graphene are loaded, without a truncation layer) shows that the reflection peak is not sufficiently sharp. After adding the truncation layer, the resonance reflection peak becomes very sharp, as indicated by the red solid line, with sharpness exceeding that of the blue solid line. As previously mentioned, the introduction of a truncation layer disrupts the periodicity of the photonic lattice by truncating one-dimensional photonic crystals on the surface, creating a local electromagnetic field on the surface of the truncation layer, resulting in local field enhancement. Considering the relationship between the defect mode and the plasmonic structure, which is similar to that formed by graphene with a truncation layer, its resonant excitation mode makes the downward reflection peak sharper and more sensitive. Even minor changes in the sensing layer can significantly affect the reflection resonance peak, providing favorable conditions for high-sensitivity sensing detection. This also lays the foundation for future research combining such structures with Bloch surface waves.

For linearly isotropic materials, their transmission characteristics can be described by the transmission matrix [32]. For simplicity, the electric field intensity of each layer in this structure can be approximated as a constant, equal to the electric field intensity on the right side of the layer. Thus, the transmission matrix of each layer is no different from the ordinary transmission matrix. Using Formula (4) to calculate the transmission matrix, the transmission matrix of the entire structure can be obtained by multiplying n transmission matrices as follows [32]:

(6) $$\left[\begin{array}{c} E_{1}\\ H_{1} \end{array}\right]=M_{1}M_{2}M_{3}\ldots M_{n-1}M_{n}\left[\begin{array}{c} E_{n+1}\\ H_{n+1} \end{array}\right]$$

where $E_{1}$ and $H_{1}$ are the electric and magnetic fields on the incident surface of the material, while $E_{n+1}$ and $H_{n+1}$ are the electric and magnetic fields on the exit surface of the material.

**Figure 3:**
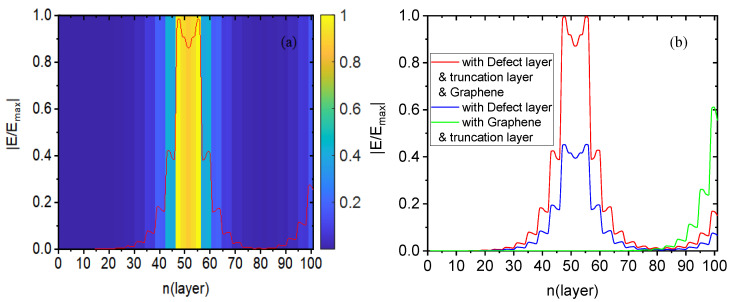
In the liquid biosensor case, (**a**) shows the electric field distribution of this structure at an angle of *θ* = 1.175°, and (**b**) presents a comparison of the electric field distribution: loaded with only a defect layer (blue solid line), loaded with a truncation layer with graphene but without a defect layer (green solid line), and loaded with a truncation layer with graphene based on the defect layer (red solid line).

Figure 3 shows the electric field intensity distribution of this structure at an angle *θ* = 1.175° where the sharp reflection peak previously mentioned is located. The width values of the structural layers in this figure are simplified, with n representing the number of layers in the structure. Here, n is used to calculate the electric field intensity from the transmission layer (n = 0) to the incident layer (n = 101), with a defect layer positioned at n = 52. From Figure 3a, two peaks of electric field intensity are observed around the defect layer, with the defect layer situated between these two peaks, forming a small valley. This indicates strong confinement of the electric field near the defect layer, and a decrease in electric field intensity is observed at further distances from the defect layer, with a decrease in intensity at greater distances, confirming the formation of the defect mode. To observe this more clearly, we use color bars to indicate the electric field intensity, where a blue color bar represents lower intensity and a yellow one represents higher intensity. The defect layer displays yellow color bars, signifying enhanced light–matter interaction and a unique double-peak-with-valley phenomenon. To further illustrate the impact of the truncation layer and graphene in this structure, we have provide a comparison diagram of the electric field intensity distribution in Figure 3b. The blue solid line corresponds to the case with only a defect layer, and the peak electric field intensity near the defect layer is 2.2 × 10^4^ V/m, which represents the phenomenon of the previously mentioned defect mode. The green solid line represents the case with a graphene-loaded truncation layer but without a defect layer, exhibiting a peak near the incident layer with an electric field intensity of 1.5 × 10^9^ V/m (normalized for ease of comparison). In this case, the photonic crystal is interrupted by the truncation layer, leading to collective oscillation of incident light and graphene surface electrons, forming a locally enhanced surface wave highly localized near the incident layer. This high electric field intensity excitation provides new directions for research and technical solutions in optical fields such as optical bistability. By combining these two elements, the electric field intensity near the defect layer of this structure is greatly enhanced, reaching a peak of 4.9 × 10^4^ V/m (as shown by the solid red line in Figure 3b), thereby further promoting light–matter interaction.

**Figure 4:**
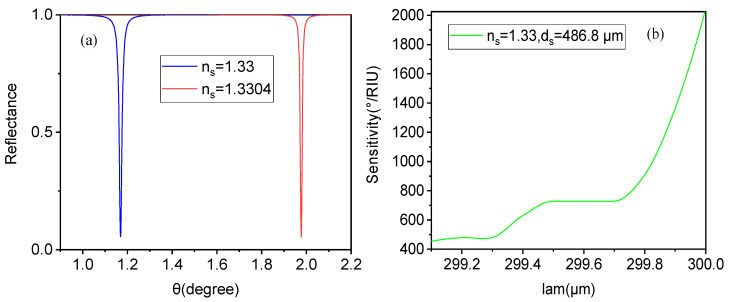
In the liquid biosensor case, (**a**) shows the relationship between reflectance and the incident angle for different sensing layers, and (**b**) illustrates the influence of variations in the working wavelength (λ) on the biosensor.

First of all, we analyzed and discussed the case of an aqueous solution containing biomolecules, assuming an initial refractive index of the liquid of $n_{s}$ = 1.33. Next, we examined the sensitivity characteristics of the entire sensor structure, assuming a refractive index change of $\Delta n_{s}$ = 0.0004 in the sensing layer caused by interactions between biomolecules in the external environment. From Figure 4a, we observed the relationship between reflectance and incident angle for different sensing layers in the aqueous solution. When the refractive index of the sensing layer of the aqueous solution is $n_{s}$ = 1.33, a sharp resonance reflection peak appears at 1.175°, consistent with the situation in Figure 2. Assuming that external environment changes caused the refractive index of the aqueous solution to become $n_{s}$ = 1.3304 due to subtle biological characteristic changes, the reflection peak of the structure shifted to a higher angle by more than 0.8°. According to the sensitivity calculation formula, the sensitivity of the biosensor at this point exceeded 2000°/RIU. This means that the reflection peak based on resonance excitation is significantly affected by structural or material parameters, making it highly suitable for high-sensitivity sensing and measurement of environmental changes. Additionally, based on the parameters in Figure 4a, the FOM value of the sensor in this case was calculated as 22,500 RIU^−1^ using the FOM expression from reference [22]. This is because the resonance peak generated by the defect mode and the truncation layer with graphene produce a very sharp reflectivity curve with a low full width at half maxima (FWHM), resulting in a high FOM for this structure. A high FOM indicates strong resolution and excellent performance of the biosensor. We also conducted adjustment tests using varying values for the working wavelength (λ), graphene Fermi level, and thickness of the truncation layer in this structure. The Fermi energy of graphene and the thickness of the truncation layer have little effect on the sensitivity but do influence the reflectivity of the reflection resonance peak, i.e., its sharpness. As shown in Figure 4b, the sensitivity increases significantly with an increasing working wavelength (λ). Nevertheless, the working wavelength cannot be increased indefinitely: on the one hand, increasing the working wavelength causes deformation in the reflection resonance peak; on the other hand, due to the close correlation between adjusting parameters and the positions of resonance excitation with the defect mode in the sensing layer, increasing the working wavelength gradually eliminates the reflection resonance peak, weakening the sensing characteristics of the overall structure. Here, a working wavelength of 300 μm was chosen.

**Figure 5:**
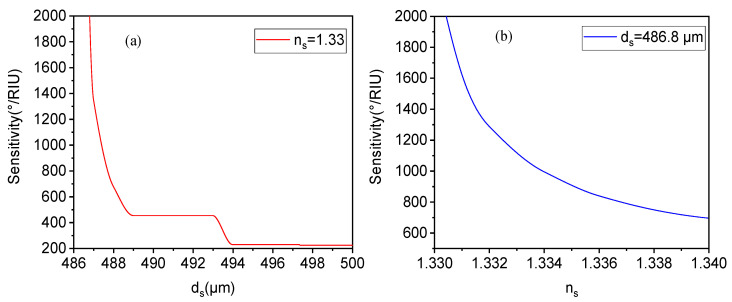
In the case of a liquid biosensor, the influence of changes in the thickness $d_{s}$ (**a**) and refractive index $n_{s}$ (**b**) of the sensing layer on its sensitivity.

As mentioned earlier, the defect layer introduces a sensing medium. In order to measure its impact on this structure, as shown in Figure 5, it is necessary to study and analyze the relationship between the parameters of the sensing layer and the sensitivity of this structure. From Figure 5a, it can be seen that there is a significant monotonic decreasing relationship between the thickness of the sensing layer and the sensitivity of the biosensor, meaning that smaller sensor thickness values can improve sensitivity. However, thickness cannot be minimized indefinitely, as this imposes higher requirements on the preparation precision and processing technology of biosensors. Additionally, due to the close relationship between the thickness of the sensing layer and the position of resonance excitation with a defect mode, adjusting the defect layer weakens the characteristics of the defect mode, leading to reduced sensing performance and the gradual disappearance of the reflection resonance peak. Thus, we only plotted the curve of the thickness above $d_{s}$ = 486.8 μm of the sensing layer. From Figure 5b, as the refractive index of the sensing layer changes, the sensitivity is greatly affected. When the refractive index of the sensing layer increases, the sensitivity gradually decreases, forming an attenuation state. The refractive index in aqueous solution sensing is typically around 1.33; for example, the refractive index of methanol is 1.3290, that of glucose is 1.345, and that of proteins is usually between 1.34199 and 1.34275 (e.g., milk whey protein). When the refractive index of the liquid changes by 0.01 relative to water, the sensitivity exceeds 600°/RIU and still has high sensitivity characteristics, indicating relatively slow sensitivity attenuation. Therefore, even though biological detection occurs in aqueous environments with a refractive index near 1.33, the biosensor can still usually meet high sensitivity requirements.

**Figure 6:**
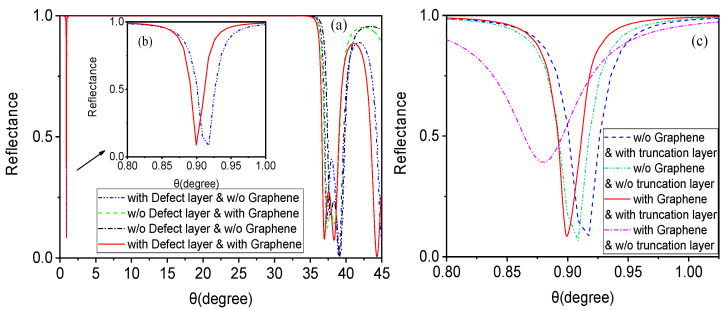
Influence of the defect layer and graphene on resonance excitation in the application case of the structure as a gas biosensor. (**a**) Comparison chart of reflectance curves with incident angle for four different states: ① loaded with a defect layer but without graphene (blue dashed line), ② loaded with graphene but without a defect layer (green dashed line), ③ loaded with neither graphene nor a defect layer (black dashed line), ④ loaded with both graphene and a defect layer (red solid line). (**b**) Enlarged partial view of reflectance curves. (**c**) The influence of the truncation layer with graphene on the curves.

Furthermore, we expanded and discussed the biosensing applications of the structure in Figure 1 to meet the needs of gases biosensing through appropriate parameter selection. In the previous liquid biosensing research, we only modified the refractive index and thickness of the sensing layer to $n_{s}$ = 1.0 and $d_{s}$ = 359.6 μm, respectively, with the other parameters unchanged. This resulted in the comparison of reflectance curves shown in Figure 6, which presents the relationship between the reflectance and incident angle for gas biosensors in four states. Figure 6a separately shows the impact of the defect layer and graphene on the reflection curve of this structure (with the truncation layer unchanged). Figure 6b provides an enlarged partial view of the reflectance curves. From this curve, it is evident that when gas serves as the medium in the sensing layer, the composite structure of one-dimensional photonic crystals embedded with a defect layer and graphene with a truncation layer generates asymmetric reflection resonance peaks corresponding to the resonance excitation. As shown in Figure 6c, when only a defect mode is loaded, the deformation of the reflection peak is very noticeable (as shown by the green dash-dotted line and the blue dashed line). However, loading the truncation layer with graphene on the basis of the defect mode not only improves the deformation of the reflection peak but also makes it sharper (as shown by the red solid line). Similarly to liquids, this reflection resonance peak is highly sensitive to small changes in the refractive index and thickness of the sensing layer.

**Figure 7:**
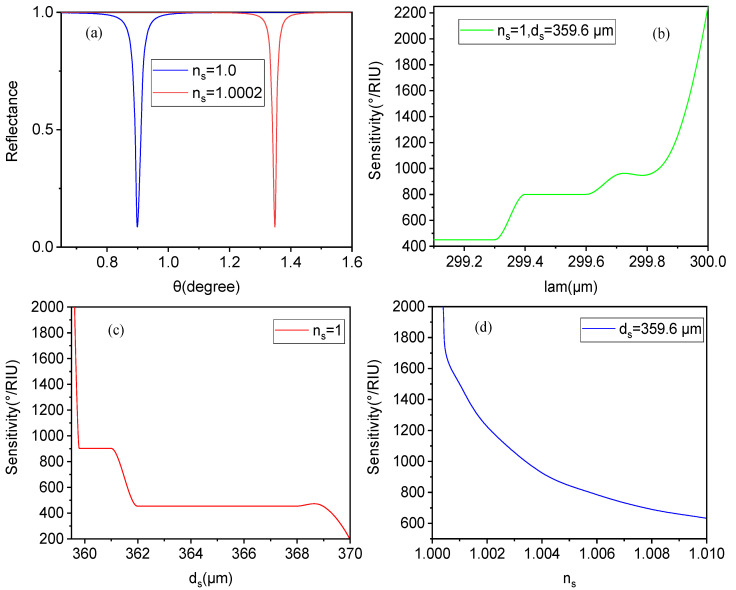
In the gas biosensor case, (**a**) shows the relationship between the reflectance and incident angle for different sensing layers, (**b**) illustrates the influence of working wavelength (λ) on the biosensor, (**c**) presents the influence of thickness $d_{s}$ on the biosensor, and (**d**) shows the influence of the refractive index $n_{s}$ of the sensing layer on the sensitivity of the biosensor.

From Figure 7a, assuming that the change rate of the refractive index is 0.0002 for gas sensing, the reflection resonance peak shifts to a higher angle by 0.8°. According to the sensitivity calculation formula, the sensitivity of the sensor exceeds 2000°/RIU, and the FOM value of 22,500 RIU^−1^ can be calculated in this case. Similarly to aqueous solutions, as the working wavelength (λ) increases, the sensitivity of the gas biosensor gradually and significantly increases, as shown in Figure 7b. Additionally, the Fermi energy level of graphene and the thickness of the truncation layer have little effect on sensitivity, but do influence the reflectivity of the reflection resonance peak, i.e., the sharpness of the reflection peak. From Figure 7c, it can be seen that the sensitivity changes with adjustments to the thickness of the sensing layer: as the thickness of the sensing layer increases, the sensitivity of the gas biosensor decreases, and vice versa. This behavior is similar to that observed in aqueous solutions. From Figure 7d, it can be seen that as the refractive index of the sensing layer changes, the sensitivity is greatly affected. When the refractive index of the sensing layer increases, the sensitivity gradually decreases, resulting in an attenuation state. In gas sensing, the refractive index is generally slightly above 1.0; for example, the refractive index of carbon monoxide is 1.000338, that of carbon dioxide is 1.000449, that of ammonia is 1.000376, that of hydrogen sulfide is 1.000634, and that of methane is 1.000444. When the change in the refractive index of the gas reaches 0.01 relative to air, the sensitivity still exceeds 600°/RIU, meaning that the biosensor retains its high sensitivity characteristics, indicating relatively slow sensitivity attenuation. Thus, the sensor generally meets high sensitivity requirements for biological detection in gas environments with a refractive index close to 1.0.

Finally, the biosensor structure proposed in this paper was compared with other biosensor structures, as shown in Table 1. In order to achieve high sensitivity and a high FOM, many schemes presented in other publicly published biosensing papers demonstrate varying sensing performance [33,34]. The design in this paper is based on a DBR structure, which is a typical example of 1D PC known for its ease of manufacturing and design [35]. Additionally, the simplicity of depositing the multi-layer stacks, the direct optical excitation using both TE light and TM light, and its high FOM mode demonstrate this structure’s potential for interesting practical applications [36] and also provide possibilities for the experimental testing and preparation of this structure. Overall, the structure proposed in this paper offers superior sensitivity, a better FOM, and a simpler design.

## 4. Conclusions

In summary, we propose a highly sensitive terahertz biosensor with a symmetrical Bragg reflector multi-layer hybrid structure. In this composite structure, a defect mode formed by photonic crystals and graphene with a truncation layer excites the reflection resonance peak. The sharpness of this resonance peak provides conditions for achieving high-sensitivity refractive index sensing. By optimizing relevant parameters, this structure enables biosensing in solutions and gases. Through structural optimization and parameters adjustment, a refractive index sensitivity greater than 2000°/RIU and an FOM value of 22,500 RIU^−1^ can be achieved in both liquid and gas sensors using this structure, demonstrating the high sensitivity of these biosensors. Additionally, when the refractive index of a sensing medium such as a solution changes to 0.01 relative to water (similarly for a gas sensing medium), the sensitivity of this structure remains above 600°/RIU, highlighting its broad applicability and slow sensitivity attenuation characteristics. Furthermore, the sensing characteristics of this structure are highly responsive and sensitive to the refractive index and thickness of the sensing medium. The sensing scheme features a simple structure, low processing requirements, high sensitivity, and a high FOM. Thus, this micro/nanostructure holds potential application value in the field of biosensing.

## Figures and Tables

**Table 1:** Comparison of refractive index sensing schemes for different mechanisms and structures.

Ref.	Mechanism	Structure	Sensitivity	FOM (RIU^−1^)	Frequency Range
[22]	OTSs sensor	Graphene–Bragg reflector structure	407.36°/RIU	65	THz
[21]	OTSs sensor	Graphene–Bragg reflector structure	517.9°/RIU	222.9	THz
[37]	SPR sensor	Otto structure	147°/RIU	/	THz
[38]	SPR sensor	Kretschmann structure	257°/RIU	45	Visible light
[39]	BSW sensor	TMDC–Bragg reflector structure	231°/RIU	48,250	Near-Infrared
[23]	Mode coupling sensor	Graphene–Bragg reflector structure (with a defect layer)	1085°/RIU	8482	THz
[24]	Mode coupling sensor	Bds–Bragg reflector structure (with a defect layer)	1022°/RIU	/	THz
This work	Defect mode-based coupling sensor	Graphene–Bragg reflector structure (with a defect layer)	2025°/RIU	22,500	THz

## Data Availability

The data presented in this study are available on request from the corresponding author.

## References

[1] Buerk D.G. (1995). Biosensors: Theory and Applications.

[2] Yang Y.P., Xu D.Q., Zhang W.L. (2018). High-sensitivity and label-free identification of a transgenic genome using a terahertz metabiosensor. Opt. Express.

[3] Nooke A., Beck U., Hertwig A., Krause A., Krüger H., Lohse V., Negendank D., Steinbach J. (2010). On the application of gold based SPR sensors for the detection of hazardous gases. Sens. Actuators B Chem..

[4] Fernández F., Hegnerová K., Piliarik M., Sanchez-Baeza F., Homola J., Marco M. (2010). A label-free and portable multichannel surface plasmon resonance immunosensor for on site analysis of antibiotics in milk samples. Biosens. Bioelectron..

[5] Singh V.V., Gupta G., Batra A., Nigam A.K., Boopathi M., Gutch P.K., Tripathi B.K., Srivastava A., Samuel M., Agarwal G.S. (2012). Greener Electrochemical Synthesis of High Quality Graphene Nanosheets Directly from Pencil and its SPR Sensing Application. Adv. Funct. Mater..

[6] Verma R., Gupta B.D. (2013). Fiber optic SPR sensor for the detection of 3-pyridinecarboxamide (vitamin B3) using molecularly imprinted hydrogel. Sens. Actuators B Chem..

[7] Zhang N.M.Y., Li K.W., Shum P.P., Yu X.C., Zeng S.W., Wu Z.F., Wang J.Q., Yong K.T., Wei L. (2017). Hybrid Graphene/Gold Plasmonic Fiber-Optic Biosensor. Adv. Mater. Technol..

[8] Konopsky V.N., Karakouz T., Alieva E.V., Vicario C., Sekatskii S.K., Dietler G. (2013). Photonic Crystal Biosensor Based on Optical Surface Waves. Opt. Express.

[9] Lo S.M., Hu S., Gaur G., Kostoulas Y., Weiss S.M., Fauchet P.M. (2017). Photonic crystal microring resonator for label-free biosensing. Opt. Express.

[10] Farrera C., Andón F.T., Feliu N. (2017). Carbon Nanotubes as Optical Sensors in Biomedicine. ACS Nano.

[11] Ahmadivand A., Gerislioglu B., Ahuja R., Mishra Y.K. (2020). Terahertz plasmonics: The rise of toroidal metadevices towards immunobiosensings. Mater. Today.

[12] Wang Y.L., Han Z.H., Du Y., Qin J.Y. (2021). Ultrasensitive terahertz sensing with high-Q toroidal dipole resonance governed by bound states in the continuum in all-dielectric metasurface. Nanophotonics.

[13] Wu L.M., Guo J., Xu H.L., Dai X.Y., Xiang Y.J. (2016). Ultrasensitive biosensors based on long-range surface plasmon polariton and dielectric waveguide modes. Photon. Res..

[14] Fouad S., Sabri N., Jamal Z.A.Z., Poopalan P. (2016). Enhanced Sensitivity of Surface Plasmon Resonance Sensor Based on Bilayers of Silver-Barium Titanate. J. Nano Electron. Phys..

[15] Zhao Y.T., Gan S.W., Wu L.M., Zhu J.Q., Xiang Y.J., Dai X.Y. (2020). GeSe nanosheets modified surface plasmon resonance sensors for enhancing sensitivity. Nanophotonics.

[16] Wu L., You Q., Shan Y., Gan S., Zhao Y., Dai X., Xiang Y. (2018). Few-layer Ti_3_C_2_Tx MXene: A promising surface plasmon resonance biosensing material to enhance the sensitivity. Sens. Actuators B Chem..

[17] Ouyang Q.L., Zeng S.W., Jiang L., Hong L.Y., Xu G.X., Dinh X.-Q., Qian J., He S.L., Qu J.L., Coquet P. (2016). Sensitivity Enhancement of Transition Metal Dichalcogenides/Silicon Nanostructure-based Surface Plasmon Resonance Biosensor. Sci. Rep..

[18] Wang C., Quan Q., Kita S., Li Y., Tian Y., Huang Y., Lončar M. (2015). Single-nanoparticle detection with slot-mode photonic crystal cavities. Appl. Phys. Lett..

[19] Yang D., Wang C., Ji Y. (2016). Silicon on-chip 1D photonic crystal nanobeam bandstop filters for the parallel multiplexing of ultra-compact integrated sensor array. Opt. Express.

[20] Jena S., Tokas R., Thakur S., Udupa D. (2020). Thermally tunable terahertz omnidirectional photonic bandgap and defect mode in 1D photonic crystals containing moderately doped semiconductor. Phys. E Low-Dimens. Syst. Nanostruct..

[21] Ye Y.Y., Xie M.Z., Tang J., Ouyang J.X. (2019). Highly sensitive and tunable terahertz biosensor based on optical Tamm states in graphene-based Bragg reflector. Results Phys..

[22] Tang J., Ye Y.Y., Xu J., Zheng Z.W., Jin X.L., Jiang L.Y., Jiang J., Xiang Y.J. (2020). High sensitivity terahertz biosensor based on optical Tamm states with graphene. Nanomaterials.

[23] Liu Y.M., Zheng Q.W., Yuan H.X., Wang S.P., Yin K.Q., Dai X.Y., Zou X., Jiang L.Y. (2021). High Sensitivity Terahertz Biosensor Based on Mode Coupling of a Graphene/Bragg Reflector Hybrid Structure. Biosensors.

[24] Bao Y.W., Ren M.J., Ji C.P., Dong J., Jiang L.Y., Dai X.Y. (2022). Terahertz Biosensor Based on Mode Coupling between Defect Mode and Optical Tamm State with Dirac Semimetal. Biosensors.

[25] Deng X., Tang H., Jiang J. (2014). Recent progress in graphene-material-based optical sensors. Anal. Bioanal. Chem..

[26] Xiao S., Zhu X., Li B.-H., Mortensen N.A. (2016). Graphene-plasmon polaritons: From fundamental properties to potential applications. Front. Phys..

[27] Descrovi E. (2008). Near-field imaging of Bloch surface waves on silicon nitride one-dimensional photonic crystals. Opt. Express.

[28] Zheng Q.W., Liu Y.M., Lu W.G., Dai X.Y., Tian H.S., Jiang L.Y. (2021). Theoretical Model for a Highly Sensitive Near Infrared Biosensor Based on Bloch Surface Wave with Dirac Semimetal. Biosensors.

[29] Jiang L.Y., Tang J., Xu J., Zheng Z.W., Dong J., Guo J., Qian S.Y., Dai X.Y., Xiang Y.J. (2019). Graphene Tamm plasmon-induced low-threshold optical bistability at terahertz frequencies. Opt. Mater. Express.

[30] Zhan T.R., Shi X., Dai Y.Y., Liu X.H., Zi J. (2013). Transfer matrix method for optics in graphene layers. J. Phys. Condens. Matter..

[31] Maharana P.K., Jha R., Palei S. (2014). Sensitivity enhancement by air mediated graphene multilayer based surface plasmon resonance biosensor for near infrared. Sens. Actuators B Chem..

[32] Xiang Y.J., Zhu J.Q., Wu L.M., You Q., Ruan B.X., Dai X.Y. (2018). Highly Sensitive Terahertz Gas Sensor Based on Surface Plasmon Resonance With Graphene. IEEE Photon. J..

[33] Polo J.A., Lakhtakia A. (2011). Surface electromagnetic waves: A review. Laser Photon. Rev..

[34] Kar C., Jena S., Udupa D.V., Rao K.D. (2023). Tamm plasmon polariton in planar structures: A brief overview and applications. Opt. Laser Technol..

[35] Lova P., Manfredi G., Comoretto D. (2018). Advances in Functional Solution Processed Planar 1D Photonic Crystals. Adv. Opt. Mater..

[36] Wang B., Yu P., Wang W., Zhang X., Kuo H.-C., Xu H., Wang Z.M. (2021). High-Q Plasmonic Resonances: Fundamentals and Applications. Adv. Opt. Mater..

[37] Sun P., Wang M., Liu L., Jiao L., Du W., Xia F. (2019). Sensitivity enhancement of surface plasmon resonance biosensor based on graphene and barium titanate layers. Appl. Surf. Sci..

[38] Goyal A.K., Saini J. (2020). Performance analysis of Bloch surface wave-based sensor using transition metal dichalcogenides. Appl. Nanosci..

[39] He M., Li T., Huo K., Zhang J., Wu F., Yin C. (2023). Low-threshold bistable absorption in asymmetrical one-dimensional photonic crystals containing Weyl semimetal defects. Eur. Phys. J. B.

